# Improvement and use of CRISPR/Cas9 to engineer a sperm-marking strain for the invasive fruit pest *Drosophila suzukii*

**DOI:** 10.1186/s12896-019-0588-5

**Published:** 2019-12-05

**Authors:** Hassan M. M. Ahmed, Luisa Hildebrand, Ernst A. Wimmer

**Affiliations:** 1 Department of Developmental Biology, Johann-Friedrich-Blumenbach-Institute of Zoology and Anthropology, Göttingen Center for Molecular Biosciences, Georg-August-University Göttingen, 37077 Göttingen, Germany; 2 Department of Crop Protection, Faculty of Agriculture-University of Khartoum, P.O. Box 32, 13314 Khartoum, Khartoum North Sudan

**Keywords:** Cherry vinegar fly, Insect transgenesis, Molecular entomology, Pest management, Spotted Wing *Drosophila*

## Abstract

**Background:**

The invasive fruit pest *Drosophila suzukii* was reported for the first time in Europe and the USA in 2008 and has spread since then. The adoption of type II clustered regularly interspaced short palindromic repeats (CRISPR)/CRISPR-associated (Cas) as a tool for genome manipulation provides new ways to develop novel biotechnologically-based pest control approaches. Stage or tissue-specifically expressed genes are of particular importance in the field of insect biotechnology. The enhancer/promoter of the spermatogenesis-specific *beta-2-tubulin* (*β2t*) gene was used to drive the expression of fluorescent proteins or effector molecules in testes of agricultural pests and disease vectors for sexing, monitoring, and reproductive biology studies. Here, we demonstrate an improvement to CRISPR/Cas-based genome editing in *D. suzukii* and establish a sperm-marking system.

**Results:**

To improve genome editing, we isolated and tested the *D. suzukii* endogenous promoters of the small nuclear RNA gene *U6* to drive the expression of a guide RNA and the *Ds heat shock protein 70* promoter to express *Cas9*. For comparison, we used recombinant Cas9 protein and in vitro transcribed gRNA as a preformed ribonucleoprotein. We demonstrate the homology-dependent repair (HDR)-based genome editing efficiency by applying a previously established transgenic line that expresses *DsRed* ubiquitously as a target platform. In addition, we isolated the *Ds_β2t* gene and used its promoter to drive the expression of a red fluorescence protein in the sperm. A transgenic sperm-marking strain was then established by the improved HDR-based genome editing.

**Conclusion:**

The deployment of the endogenous promoters of the *D. suzukii U6* and *hsp70* genes to drive the expression of *gRNA* and *Cas9*, respectively, enabled the effective application of helper plasmid co-injections instead of preformed ribonucleoproteins used in previous reports for HDR-based genome editing. The sperm-marking system should help to monitor the success of pest control campaigns in the context of the Sterile Insect Technique and provides a tool for basic research in reproductive biology of this invasive pest. Furthermore, the promoter of the *β2t* gene can be used in developing novel transgenic pest control approaches and the CRISPR/Cas9 system as an additional tool for the modification of previously established transgenes.

## Background

Native to East Asia [1], the cherry vinegar fly *D. suzukii*, also known as the Spotted Wing *Drosophila* (SWD) was reported for the first time in Europe, Spain and Italy, and the mainland USA in California in 2008 [1–3]. The pest has since then expanded its geographic distribution to include all of Europe as reported by the European Plant Protection Organization [2]. In the USA, the situation is as severe as in Europe. Four years after its first invasion in California, the SWD has been reported in more than 41 states [4]. By now, this invasive insect pest has also been reported further down in South America: for the first time between the years 2012 and 2013 in Brazil [5] and more recently also in Argentina in four localities [6].

The devastating fruit pest *D. suzukii* infests mainly soft-skinned as well as stone fruits with a wide host range spanning cultivated and wild plants [7]. In contrast to other *Drosophila* spp., the SWD is armoured with a sharp serrated ovipositor, which allows it to infest ripening and not only overripe or rotten fruits [8]. Earlier studies have shown that economic impact due to the infestation is in the order of millions of US dollar [9, 10]. Current control efforts mainly rely on heavy application of insecticides [11, 12], which is on the one hand not compatible with organic farming and prone to rapid emergence of insecticide resistance owning to the short generation time of this fly. And on the other hand, it is not safe, as the time between onset of infestation and harvest is very short and does not allow for a sufficiently long period post pesticide application. Other control strategies include the use of natural enemies such as parasitoids, predators, or pathogens [13], netting to cover the plants [14], and good cultural practices to minimise the source of infestation [15]. The sterile Insect technique (SIT) presents itself as an additional safe and effective pest management strategy. It provides a species-specific, environmentally sound pest control approach [16] and is compatible with other pest control strategies in integrated pest management (IPM) programs. The system has been proposed more than half a century ago and was used to successfully eradicate the tsetse fly from Zanzibar as well as the screw worm from Libya and the USA [17, 18]. It encompasses mass production of the target insect, removal of the females, and sterilization of males by ionizing radiation prior to release [16]. Using transposon-based germline transformation, many transgenic strategies have been developed to overcome some of the drawbacks of classical SIT. A transgene-based embryonic lethality system was developed for several dipterans including the model *D. melanogaster* and the cosmopolitan fruit pest *Ceratitis capitata* [19, 20]. The system relies on the ectopic expression of a pro-apoptotic gene during early embryonic stages, which leads to cell death and hence reproductive sterility [19]. The same system has also been used for sexing, when the embryonic lethality was rendered female-specific by making use of the sex-specifically spliced intron of the *transformer* gene, which allows for elimination of females at the embryonic stage [20–22]. Furthermore, for monitoring the competitiveness of released males, sperm-marking systems were developed for a number of pest insects and diseases vectors by driving the expression of fluorescent protein during spermatogenesis [23–26].

Recently, a revolution in genome engineering was started by the application of the CRISPR/Cas system, which stands for type II clustered regularly interspaced short palindromic repeats CRISPR/CRISPR-associated. Respective sequences were first observed in bacterial genomes in 1987 [27]. Two decades later, researchers found an association between these repeated sequences and resistance of bacteria to bacteriophages [28] and showed that the bacteria use this system as an adaptive defence mechanism against invading DNA elements [29]. The system consists of the Cas9 effector endonuclease, the CRISPR RNA (*crRNA*), which confers specificity to Cas9, and the transactivating crRNA (*tracrRNA*), which facilitates maturation of *crRNAs* and the interaction with Cas9 protein for forming active RNP complexes [30, 31]. The *crRNA* and *tracrRNA* were fused together to generate a single chimeric gRNA that facilitated the use of the system [32]. The Cas9 endonuclease can easily be programmed to target and induce DNA double strands break (DSB) by replacing the 20 nucleotides (spacer) at the 5′ of the *crRNA* with 17–20 nucleotides (nt) complementary to the target of interest. The prerequisite for the RNP complex to unwind, bind, and induce DSB in the target DNA is a proto-spacer adjacent motif (PAM) immediately downstream of the 20 nt target sequence, which is NGG in the case of the most commonly used *Sp_Cas9* from *Streptococcus pyogenes* [31]*.* Similar to other programmable endonucleases such as Zinc finger nucleases (ZFNs) and Transcription activators like nucleases (TALENs), the role of Cas9 as a genome editing tool ends with the induction of a DSB. Repairing the genome - by either homology directed repair (HDR) or by non-homologous end joining (NHEJ) - is a function of the cell own DSB repair machinery, the stage of the cell at which the DSB is induced, and the availability of homologous DNA [32]. The system has rapidly been adopted as a genome engineering tool for many model and non-model organisms including zebrafish [33], mouse [34, 35], *Drosophila* [36], mosquitoes [37, 38], and human cell lines. The CRISPR/Cas9 system has also been used to induce chromosomal translocations in embryonic stem cells [39], and to engineer new balancer chromosomes in the nematode model *Caenorhabditis elegans* [40].

In the genetics power horse *D. melanogaster*, CRISPR/Cas9 has been used and delivered in different forms: as helper plasmids, mRNA and gRNA, as well as a ribonucleoprotein complexes. Several promoters have been used to drive the expression of *Cas9* including germline-specific promoters of genes such as *nanos* and *vasa*, inducible promoters such as *heat shock protein 70* (*hsp70*), and promoters of ubiquitously expressed genes such as *Actin5C*. Systematic analysis of the three different promoters of the *small nuclear RNA (U6)* genes in *D. melanogaster* has shown that the *U6:3* promoter drives the strongest expression measured by gene editing events [41, 42].

In *Drosophila suzukii*, the CRISPR/Cas9 system has been used albeit with low efficiency to mutate the genes *white* (*w*) and *Sex lethal* (*Sxl*) using *D. melanogaster* promoters to drive the expression of *gRNA* and *Cas9* [43]. Another study reported on the use of pre-assembled a ribonucleoprotein complex (RNP) to induce mutations in the *white* gene [44]. The introduction of the mutations was in both studies based on NHEJ. The system has also been used to engineer by HDR a temperature sensitive mutation in the *Ds_transformer-2* gene (*Ds_tra-2*) that leads to sex conversion. In this study a RNP complex in combination with RNA interference against the *Ds_lig4* gene was used and an HDR frequency of 7.3% was reported [45]. Furthermore, a RNP complex has also been used in a behavioural study of *D. suzukii* to knockout the gene that encodes the odorant receptor co-receptor (Orco) by HDR-mediated mutagenesis [46].

In applied insect biotechnology, CRISPR/Cas9 has become very popular particularly in the development of insect control strategies. One possible application for the system in SIT is the development of a reproductive sterility system that targets Cas9 to induce many DSBs at defined loci during spermatogenesis. This could mimic the desired effect of ionizing radiation in generating redundant sterility and at the same time overcome the random action of radiation affecting all organs, which reduces the overall fitness of the sterile males [47].

To restrict Cas9 activity to spermatogenesis, the isolation of a tissue-specific promoter is essential. The *Drosophila β2t* gene has been shown to code for a β-tubulin, which is expressed in a tissue-specific manner during spermatogenesis [48]. Its testes-specific expression makes it a good candidate for developmental studies related to reproductive biology and male germline development as well as pest control strategies. *Dm_β2t* is a TATA-less gene, which relies on an initiator element (Inr) as a core promoter with the testes-specific expression conferred by a 14 bp activator element called *β2 Upstream Element 1* (*β2UE1*) [49]. Further elements required for the expression level are *β2UE2* at position − 25 and *β2DE1* at position + 60 [50]. Homologs of *Dm_β2t* were identified in a number of insects including *Anopheles stephensi, Aedes aegypti, Ceratitis capitata, Anstrepha suspensa, Anastrepha ludens*, and *Bacterocera dorsalis* [23–26]. The upstream regulatory sequence has been used to drive the expression of fluorescent protein in the testes, which serves as a strategy for sex separation as well as for monitoring released males in SIT. In the major malaria vector *Anopheles gambiae*, the promoter of the *β2t* gene was used to drive the expression of the homing endonuclease *I-Ppol* during spermatogenesis. *I-Ppol* is a highly specific Homing Endonuclease Gene (HEG), which targets and cuts a conserved sequence within the *rDNA* on the X chromosome and thereby leads to X-chromosome shredding leaving mostly Y-chromosome bearing sperm functional, which results in sex-ratio distortion [51].

In this study, we present an improved CRISPR/Cas9-based genome engineering system for the invasive fruit pest *D. suzukii* and its application to edit a transgenic line generated using *piggyBac* germline transformation. Moreover, we report on the use of this editing system to generate a *D. suzukii* sperm marking line based on the *Ds_β2t* promoter driving the expression of *DsRed* in the testes*.*

## Results

### Improvement on CRISPR/Cas9 genome editing in *Drosophila suzukii*

In order to improve on the HDR-mediated genome editing based on CRISPR/Cas9-induced DSBs, we isolated endogenous polymerase II (*hsp70* gene) and polymerase III promoters (*U6* genes) from *D. suzukii* to drive *Cas9* or *gRNAs*, respectively. Searching for homologs of the *D. melanogaster heat shock protein 70* (*hsp70*) gene, we identified the *D. suzukii Ds_hsp70* gene, cloned and sequenced 500 bp upstream of the ATG translation start codon and used this upstream sequence to drive the expression of *Cas9*.

**Fig. 1:**
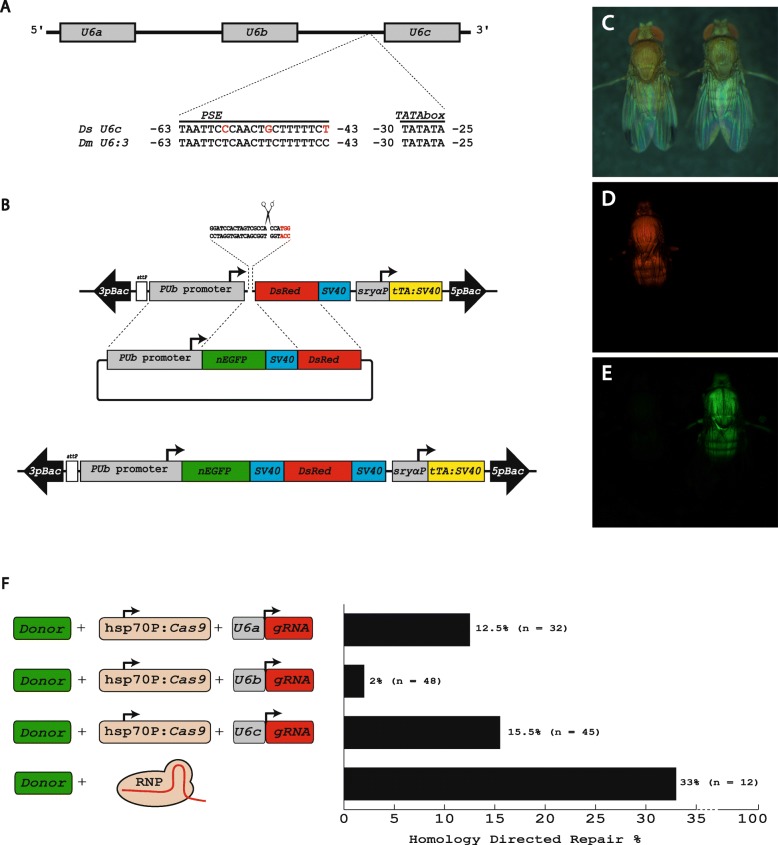
Improvement of genome editing in *D. suzukii*. **a** Three copies of the *snRNA* gene *U6* in the genome of *D. suzkuii*. The transcription from *U6* genes by *RNA pol III* is directed by the proximal sequence element *PSE* which is highly conserved between *D. suzukii* and *D. melanogaster*. **b** Scheme for HDR-based genome editing at a transgenic target platform. Sequence of the target site in the transgenic strain showing the PAM sequence in red. The scissors indicate where Cas9 induces the DSB three nucleotides upstream of the PAM. **c-e** Fluorescent marker change as the result of the HDR knock-in: images of two male flies taken with cold light (**c**), RFP fliter (**d**), and EYFP filter (**e**). **f** Comparison of *Ds U6a, U6b, U6c* promoters as well as RNP in their efficiency to promote HDR-mediated knock-ins

First attempts using PCR to isolate the *U6* genes based on *D. suzukii* genome database sequences were not successful. The presence of three tandem copies obviously rendered the assembly inaccurate. Since *D. suzukii* is a close relative to *D. melanogaster*, we then tried to isolate the *U6* locus based on synteny cloning: we amplified and sequenced a 3.7 kbp fragment encompassing the *U6* locus. We identified three *U6* genes and refer to them in 5′ to 3′ direction as *U6a*, *U6b*, and *U6c* (Fig. 1a) to distinguish them from their *D. melanogaster* equivalents.

To test the efficiency of the endogenous *hsp70* and *U6* promoters in order to drive the expression of *Cas9* and *gRNA*, respectively, for mediating HDR-based genome editing, we used the embryonic line 06_F5M2 generated by *piggyBac* germline transformation as a target platform (Fig. 1b). This driver line can be used to express the heterologous tetracycline-controlled transactivator *tTA* gene specifically at early embryonic stages due to the use of the enhancer/promoter element of the cellularization gene *Ds_sryα*. Such lines can be employed to establish conditional embryonic lethality for reproductive sterility [19, 20] or conditional female-specific embryonic lethality [21, 22, 52]. As a transgenic marker, this line expresses *DsRed* under the *D. melanogaster* promoter of the *polyubiquitin* (*PUb*) gene*.* Based on a T7EndoI assay, a functional guide targeting upstream of the *DsRed* translation start codon was identified (Fig. 1b). In a first attempt, in which donor (HMMA134), Cas9 (HMMA 056), and gRNA (HMMA104; *U6c*) plasmids were injected at concentrations of 350, 400, and 150 ng/μl, respectively, we obtained 9.5% homology directed repair (HDR) knock-in events, which we scored based on the change of the body marker from *DsRed* to *EGFP* (Fig. 1c-e). Sequencing of the knock-in junctions revealed faithful scar-less HDR events. The HDR was facilitated by the 1989 bp left homology arm (*PUb* promoter) and the 672 bp right homology arm (*DsRed*).

To compare the three promoters of the *DsU6* genes, we injected in a second attempt donor (HMMA134), *Cas9* (HMMA056), and either of the three gRNA plasmids HMMA102 (*U6a*), HMMA103 (*U6b*), or HMMA104 (*U6c*) at a concentration of 400, 400 and 250 ng/μl, respectively. This resulted in HDR events of 12.5, 2, and 15.5% for *U6a*, *U6b*, and *U6c*, respectively (Fig. 1f). Injection of a RNP complex resulted in 33% HDR events (Fig. 1f). This indicates, that at slightly higher concentrations of donor template and gRNA plasmids, we were able to obtain 15.5% knock-in events using the *U6c* promoter. The *U6b* showed the lowest performance with only 2% knock-in events, and *U6a* was intermediate with 12.5% efficiency (Fig. 1f). Interestingly, the tendency observed for the strength of the different promoters is in line with their *D. melanogaster* counterparts. The high HDR-rates of above 10% indicate that the use of the endogenous promoters allows for effective application of helper plamids instead of RNPs to induce HDR-dependent knock-ins, which represents an improvement for CRIPR/Cas9-based genome editing in *D. suzukii*.

### Isolation of the *ß2 tubulin* gene from *Drosophila suzukii*

**Fig. 2:**
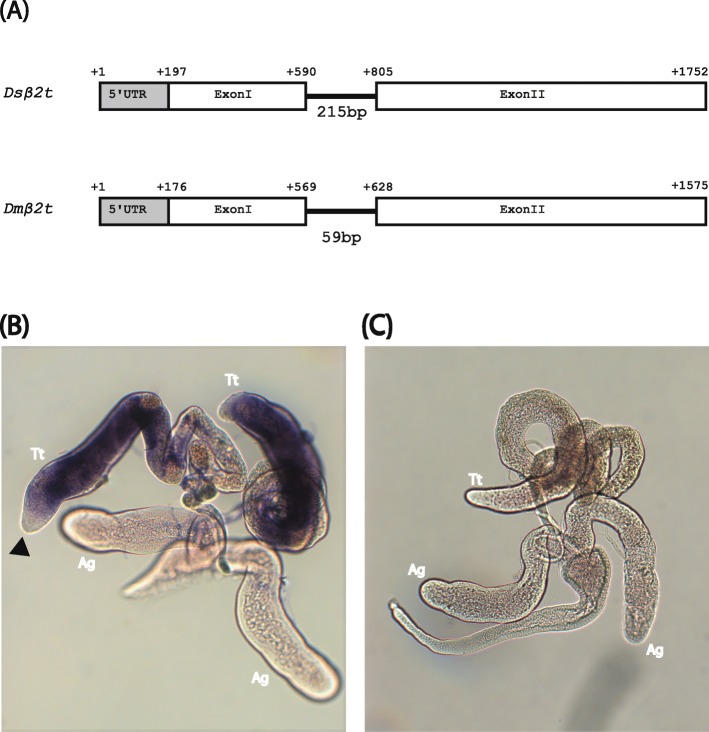
*D. suzukii β2t* gene and its expression. **a** *Ds_β2t* gene has two exons and one intron similar to *D. melanogaster*. The gene is slightly longer in *D. suzukii* due to increase in the size of the 5’UTR and the intron. The numbers indicate the first nucleotide of the respective feature relative to the first transcribed nucleotide. **b** Testes whole mount in situ hybridization using DIG labeled RNA antisense probe against *Ds_β2t* 5’UTR and exon I detects strong and testes-specific expression. The gene is not expressed at the tip of the testes (black triangle) where stem cells reside. **c** Negative control using DIG labeled sense probe shows no signs of staining. The abbreviations Tt and Ag refer to testes or the accessory glands, respectively

To be able to drive sperm-specific gene expression, we identified the *Ds_β2t* gene by homology search in the *D*. *suzukii* genome database (www.spottedwingflybase.org) using the *Dm_β2t* sequence as query. The open reading frame of the *Ds_β2t* gene from the translation start codon to the stop codon is 1341 bp, which is interrupted by a 215 bp intron. The gene has a 5’UTR of 196 bp, which demarcates the transcription start site (Fig. 2a). Conceptual translation of the *Ds_β2t* coding sequence gives rise to a protein of 446 amino acids.

To validate the testes-specific gene expression of the isolated *Ds_β2t* gene, we performed whole mount in situ hybridization on the complete reproductive tract of 3–5 day old males using DIG-labelled antisense and sense RNA probes against the *Ds_β2t* 5’UTR and exon I. These in situ hybridizations detected expression only in the testes with no expression at the apical part that consists of stem cells (Fig. 2b). No transcription was detected in the rest of the reproductive tract (Fig. 2b) or with sense RNA probe as negative control (Fig. 2c).

### Generation of a sperm-marking line of *Drosophila suzukii*

**Fig. 3:**
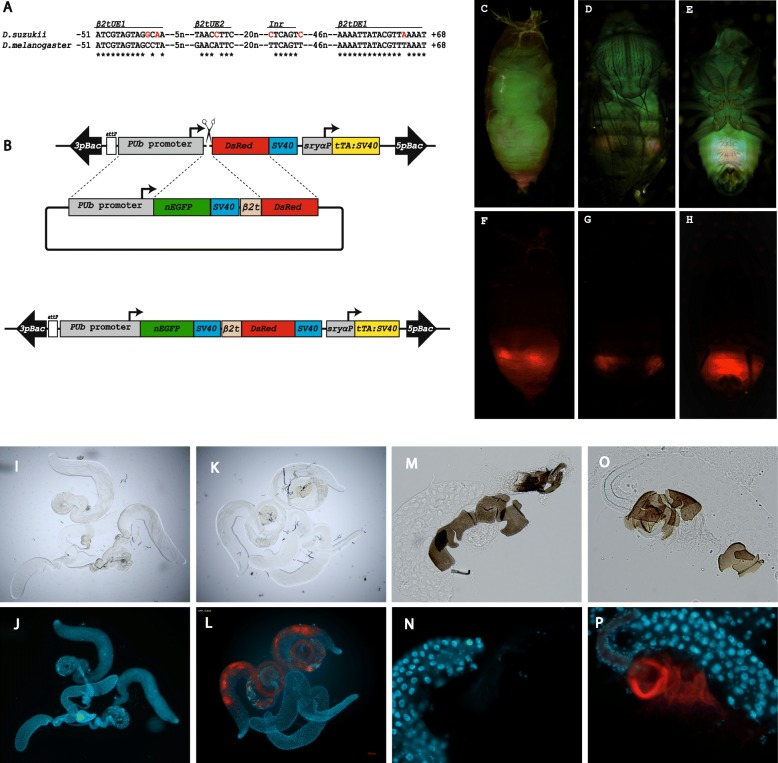
Generation of a sperm marking strain. **a** *Drosophila β2t* genes have a very short and highly conserved promoter/enhancer region with a 14 bp upstream element (*β2tUE1*) that confers testes-specific expression while the other indicated elements play quantitative roles. **b** Scheme for HDR knock-in of the repair template having *EGFP:SV40* and *β2t* promoter fused to *DsRed*. **c**-**h** Result of the HDR knock-in: images of Pupae (**c**, **f**) as well as adult males in dorsal (**d**, **g**) or ventral view (**e**, **h**) taken with GFP-LP (**c**-**e**) or RFP (**f**-**h**) filters, respectively. Compared to wild type (**i, j**), the testes of the knock-in males show strong expression of *DsRed* under control of the *β2t* promoter (**k, l**). In contrast to wild type females mated to wild type males (**m, n**), the fluorescent sperm can also be detected in the storage organ (spermatheca) of wild type females mated to the transgenic sperm-marked strain (**o, p**). **i**, **k**, **m**, **o** images were taken under bright field, and **j**, **l**, **n**, **p** are composites of images made of the same objects using a DAPI and a DsRed filter

To identify the necessary upstream and downstream regulatory elements driving sperm-specific gene expression, we compared the *D. suzukii β2t* sequence with the characterized counterpart in *D. melanogster*. The 14 bp upstream activator element *β2tUE1* that confers testes specificity to the *β2t* gene was found at the exact position − 51 to − 38 relative to the transcription start site with a C > G exchange at position − 41 and a T > A exchange at position − 39 (Fig. 3a). A second upstream regulatory element, *β2tUE2*, which is not involved in specificity but its overall activity, was identified at position − 32 to − 25 with a G > T exchange at position − 32 and an A > C exchange at position − 28. Another element that functions as a TATAAA-box in TATA-less promoter is the 7 bp initiator sequence encompassing the transcription start, which was identified − 3 to + 4 with the first and last nucleotide differing from *D. melanogaster* (Fig. 3a). A further element involved in *β2t* promoter function is the *β2tDE1* element that is highly conserved and lies relative to the transcription start site at position + 51 to + 68 (Fig. 3a).

To examine whether the 51 bp upstream regulatory element plus 196 bp 5’UTR (− 51 to + 196) drives strong testes-specific gene expression, we fused this 247 bp enhancer/promoter fragment of the *Ds_β2t* gene to *DsRed.T3* (Fig. 3b) and performed an HDR-based knock-in into the *D. suzukii* embryonic *piggyBac* line 06_F5M2, which we had used before as target platform (Fig. 3b). The repair template consisted in this case of *EGFP* fused to the *PUb* promoter followed by *SV40* 3’UTR and the 247 bp *Ds_β2t* promoter fused to *DsRed.T3* (Fig. 3b). The HDR-based knock-in resulted with 13.3% efficiency. One of the resulting *D. suzukii* lines, 134M16M2, showing a ubiquitous green fluorescence and testes-specific red fluorescence (Fig. 3c-h), was moleculary characterized to confirm the proper HDR event. In this line, red fluorescent sperm could be detected in the testes (Fig. 3i-l) and males of this line transferred red fluorescent sperm to the female spermatheca (Fig. 3m-p). This line 134M16M2 thus serves as a sperm-marking line for this invasive pest insect.

## Discussion

The programmable genome editing system CRISPR/Cas9 has enabled a series of new strategies of biotechnological engineering in model and non-model organisms. Based on the objective of the study, financial resources, and availability of functional promoters, researchers can chose the best strategy for delivery of CRISPR/Cas9 components. From published literature, it can be concluded that the most efficient strategy is germline-specific transgenic expression of Cas9, followed by application of RNP-complexes, then mRNA and gRNA co-injection, and with the least efficiency helper plasmids co-injection [42, 53]. The latter, however, is the most convenient even though it requires the identification and characterization of suitable promoters.

CRISPR/Cas9 holds big promises in the field of insect biotechnology especially for the development of novel pest control strategies, such as reproductive sterility systems based on chromosome shredding [47]. To be able to engineer such strategies in *D. suzukii*, promoters that drive strong expression of gRNAs and other components are of particular importance. Inducible promoters of heat shock genes such as *D. melanogaster hsp70* and *Tribolium castaneum Tc_hsp68* have been used for a long time to conditionally express genes both transiently from a plasmid and as transgenes [54, 55].

Due to their defined transcription start site and transcription termination, the RNA *polIII* promoters of the small nuclear RNA genes (snRNA) *U6* have been widely used to express short hairpins to induce an RNA interference effect. With the development of the CRISPR/Cas9 genome editing system, such promoters gained even more popularity and have intensively been used to drive the expression of the chimeric gRNAs transiently and as transgene components from mammals to plants. *D. melanogaster* has three copies in tandem on the right arm of chromosome 3 and have the cytological map location 96A, based on which they were termed *U6:96Aa*, *U6:96Ab*, and *U6:96Ac*. The promoters of the three genes were systematically tested and the promoter of the *U6:96Ac* gene (referred to also as *U6:3*) outperforms the other two, which made it the promoter of choice among Drosophilists. Our results are consistent in this respect, as also the *Ds_U6c* promoter has the highest effectivity (Fig. 1f).

Previous reports demonstrated the functionality of the promoters of *Dm-U6:3* and *vasa* genes to drive expression of *gRNA* and *Cas9*, respectively, to target and mutate *D. suzukii w* and *Sxl* by NHEJ but with low frequency. The authors argued that this low efficiency might be attributed to the use of plasmids to drive the expression of *Cas9* and *gRNA* or their bulk crossing scheme [43]. Another study demonstrated the feasibility of using RNP-complexes to induce mutations in *D. suzukii w* by NHEJ [44]. In a more recent study, researchers used RNP-complexes to induce DSBs and were able to knock-in by HDR a mutated temperature-sensitive version of *Ds-tra2* along with a transformation marker cassette. They reported on 7.3% HDR events even though they tried to shift the cell DSB repair machinery towards HDR by co-injection of dsRNA against the *Ds_lig4* gene [45]. In our hands, using RNP complex resulted in a four times higher rate of HDR-based knock-ins. However, no direct comparison with the previous studies is possible since the target itself is different. Anyway, also our helper plasmid co-injections yielded a two times higher rate of HDR-based knock-ins, which indicates that the isolated endogenous promoters allow for an efficient application of the CRISPR/Cas system with the more convenient use of plasmid helpers. However, if the objective is to manipulate the genome and recombinant Cas9 is available, the RNP approach is probably the best option, if no transgenic lines expressing *Cas9* in the germline are available. Studies in *D. melanogaster* and mosquitoes also showed that the use of RNP-complexes always leads to better editing results compared to injection of plasmids or mRNA and in vitro transcribed gRNA.

The use of the regulatory elements (enhancer/promoter) of sex-, tissue-, or stage-specifically expressed genes to drive effector molecules in a particular sex or developmental stage is not only useful in basic research to elucidate gene function, but also in applied insect biotechnology to develop transgene-based pest control strategies. The gene *β2t* has been identified in a number of insects to be testes-specific with its activity starting at the late larval instar. The gene in *D. melanogaster* is known to code for a 446aa protein. Here, we identified the *D. suzukii* homolog that shows at the amino acid level 100% identity but not at the nucleotide level. Interestingly, the transcript structure of the *Ds_β2t* gene revealed the presence of a 215 bp intron (Fig. 2a) compared to a highly conserved intron of 57 bp in *Aedes egypti* [24], 58 bp in *Anastrepha ludens*, 59 bp in *D. melanogaster*, 60 bp *Anstrepha suspensa*, and 67 bp in *Bacterocera dorsalis* [25]. Testes whole mount in situ hybridization identified a similar expression pattern as previously obtained in *D. melanogaster* with the apical part of the testes that contains the stem cells not expressing the gene. The testes specificity of the gene is conferred by a 14 bp activator element upstream of the transcription start site called upstream element *1 β2tUE1*, which is not only contextually conserved but also spatially relative to the transcription start site and other regulatory elements. This activator element was also identified in *D. suzukii*, which shares high similarity to its *Dm_β2*t counterpart. The other elements that are quantitatively contributing to the expression of *β2t* were also identified in exactly the same positions as in *D. melanogaster* relative to each other and to the transcription start site.

The promoter of the *β2t* gene has been used to drive the expression of a fluorescent protein in mosquitoes and tephritid fruit flies [23, 24, 26], which serve as a sexing system to automate separation of males from females and also as a monitoring system for released males in the context of SIT programs. The generated sperm marking strain of *D. suzukii* proved that the 247 bp regulatory sequence made of 51 bp upstream sequence plus 196 bp leader immediately upstream of the translation start codon has the necessary elements to drive expression of effector molecules specifically in the sperm. The fluorescent sperm can also be identified stored in the spermathecae of wild type females mated to the transgenic sperm marked strain, which facilitates monitoring and allows assessment of the competitiveness of released sterile males compared to their wild type counterparts. The sperm marking system can also help in conducting reproductive biology studies that will enrich our understanding of the biology of this pest and allow us to better design pest control strategies. For example, the promoter of the *β2t* gene in *Anopheles* was used to drive the expression of an HEG that targets and shreds the X chromosome in the mosquito during spermatogenesis leading towards a Y sperm bias and as a consequence to sex ratio distortion, which eventually can lead to a population collapse [51].

## Conclusion

We obtained improved usability of the CRISPR/Cas9 gene editing in *D. suzukii* compared to previous reports [43–45] by the employment of helper plasmids that contain endogenous promoters of the *U6* and *hsp70* genes to drive the expression of *gRNA* and *Cas9*, respectively. Moreover, we show that the CRISPR/Cas9 system can be used as an additional tool for the modification of previously established transgenes. The identification and cloning of the *β2t* promoter enabled us to generate a sperm-marking system in *D. suzukii*, which provides a tool for basic research in reproductive biology and should help to monitor the success of pest control campaigns in the context of SIT [23–26]. In addition, the *β2t* promoter can be used in developing novel transgenic pest control approaches [47] for this invasive pest insect.

## Methods

Unless otherwise specified, all PCR amplifications were performed using Phusion DNA polymerase and Phusion-HF buffer (New England Biolabs GmbH, D-65926 Frankfurt am Main). Routine plasmid min-preps and PCR products were purified using NucleoSpin® Plasmid and NucleoSpin® Gel and PCR Clean-up kits (Macherey-Nagel GmbH & Co., 52,355 Dueren, Germany), respectively. Plasmid vectors for microinjections were prepared using NucleoSpin® Plasmid Transfection-grade (Macherey-Nagel) or QIAGEN Plasmid Plus Midi Kit (QIAGEN GmbH, 40,724 Hilden, Germany). Primers used are listed in Additional file 1: Table S1.

### Fly strain and husbandry

All fly experiments were performed in our well-equipped safety level one (S1) laboratory, which is certified for generating and using genetically modified insects. Wild type *D. suzukii* from Italy (kindly provided by Prof. Marc F. Schetelig) as well as generated transgenic lines were reared on standard *Drosophila* food supplemented with baker yeast and kept at 25 °C throughout this study. For germline transformation, flies were transferred to *Drosophila* egg laying cages and allowed to lay eggs on apple juice agar plates with some yeast on top to increase egg laying.

### Nucleic acid isolation

Genomic DNA was isolated from a mix of adult males and females of *D. suzukii* (Italian strain) using NucleoSpin® DNA Insect (Macherey-Nagel) according to the manufacturer instructions. To generate a testes-specific cDNA library, testes of 100 males (3–4 days old) were dissected in ice cold 1X PBS and used for total RNA preparation using ZR Tissue & Insect RNA MicroPrep (Zymo Research Europe, 79,110 Freiburg) according to manufacturer instructions.

### Isolation of *DsU6* and *hsp70* genes

Based on synteny we identified *D. suzukii* the homologs of *D. melanogaster* genes *Esyt2* and *REPTOR* bordering the *U6* locus. Primer pair HM#137/138 was designed on the conserved parts of these genes and used to PCR amplify the sequence between them supposedly containing the *Ds_U6* locus, (initial denaturation temperature 98 °C 3 min followed by 35 cycles of 98 °C 30s, 72 °C 2 min 30 s). A 3.7 kbp fragment was obtained and sequenced.

To identify the *D. suzukii heat shock protein 70* (*Dshsp70*) gene, we BLASTed *D. melanogaster hsp70*Aa in the *D. suzukii* genome data base (www.spottedwingflybase.org) and compared the amino acid sequence as well as the corresponding DNA sequence individually to their *D. melanogaster* counterparts using the geneious program version 10.2.6 (Auckland, 1010, New Zealand).

### Isolation of *Dsβ2t* gene and its 5’UTR

To isolate the spermatogenesis specific *beta-2-tubulin* (*β2*t) gene of *D. suzukii*, we searched in the www.spottedwingflybase.org with the *D. melanogaster Dm_β2t* gene. A putative *Ds_ β2t* gene sharing high homology to *Dm_β2t* was PCR amplified from genomic DNA using primer pair HM#25/26 and the PCR program 98 °C for 3 min followed by 35 cycles of 98 °C 30 s, 72 °C 1 min 40 s, and 7 min final elongation at 72 °C. The amplified fragment was purified, blunt cloned into pJet1.2 vector (Thermo Fisher Scientific, 64,293 Darmstadt, Germany), and sequenced using standard primers pJet1.2_fwd and pJet1.2_rev.

Since the 5’UTR of *β2t* has some regulatory elements, whose position relative to the transcription start site and the upstream regulatory elements is highly conserved and important for correct tissue specific expression, it was imperative to isolate the 5’UTR and to identify the transcription start site. To do so, 1.7 μg of testes total RNA were used to generate a 5′ RACE-ready cDNA library using the SMARTer™ RACE cDNA amplification kit (Takara Bio Europe SAS, 78100 Saint-Germain-en-Laye, France) according to manufacturer instructions. The 5’UTR was recovered by RACE PCR using gene specific primer HM#33 and universal primer (UPM) provided with the kit using Advantage2 DNA polymerase (Takara) with the following program: 94 °C 2 min, (94 °C 30 s, 72 °C 3 min) 5X, (94 °C 30 s, 70 °C 30 s, 72 °C 3 min) 5X, (94 °C 30 s, 68 °C 30 s, 72 °C 3 min) 30X. A single prominent band was recovered, purified, cloned into pCRII (Thermo Fisher Scientific) to generate pCRII_Dsb2t_5’UTR (HMMA24), and sequenced using a standard M13 primer.

### Testes whole mount in situ hybridization

To generate DIG-labelled sense and antisense RNA probes of *Ds_β2t*, we prepared DNA templates for in vitro transcription by PCR amplification of the 5’RACE-fragment including the Sp6 or T7 promoters from pCRII_Ds *β*2t_5’UTR (HMMA24). Primer pairs HM#33/128and HM#41/127 were used respectively with the following PCR conditions: initial denaturation at 98 °C 3 min, followed by 35 cycles of 98 °C 30 s, 72 °C 50 s with a final elongation step of 7 min. RNA probes were synthesized using DIG-labelling kit (Thermo Fisher Scientific) according to manufacturer instructions using 200 ng of DNA as template in a total reaction mix of 10 μl. The reaction was allowed to proceed for 2 h at 37 °C followed by Turbo DNaseI treatment (Thermo Fisher Scientific) for 15 min to remove template DNA. Two microliter of 0.2 M EDTA was used to inactivate the reaction. Sense and antisense probes were precipitate and resuspended in 100 μl RNA resuspension buffer (5:3:2 H2O: 20X SSC: formaldehyde) and stored at − 80 °C.

Testes of 3–5 days old males were dissected in ice cold 1X Phosphate buffered saline (PBS) and fixed in PBF-tween (4% formaldehyde and 0.1% tween 20 in 1X PBS) for 20 min at room temperature. In situ hybridization was performed according to an established protocol [56] with inclusion of dehydration steps according to Zimmerman et al. [57].

### Plasmid construction

To generate plasmid HMMA006, 300 bp upstream of *Ds_sryα* plus 50 bp 5’UTR sequence were PCR amplified using primer pair HM#23/24 introducing *AgeI/NheI* cut sites respectively and cloned into *AgeI/NheI* cut site of KNE007 [58] upstream of *tTA* CDS replacing the *Dm_β2t* promoter. Description of the *Ds_sryα* gene and its cloning will be described elsewhere (Ahmed et al.)

To generate pSLaf_*T7-BbsI-BbsI-ChiRNA*_af (HMMA034) for in vitro transcription of gRNAs, annealed oligos HM#55/56 generating T7 promoter and 2X *Bbs*I restriction sites were cloned into *Bbs*I/*Hind*III digested plasmid p*U6-chiRNA* (Addgene: #45946) giving rise to HMMA033. Next, the *Hind*III/*Sac*I *T7-BbsI-BbsI-chiRNA* fragment from HMMA033 was cloned into pSLaf1180af [59] *Hind*III/*Sac*I cut sites.

To generate plasmids *pDsU6a-BbsI-BbsI-chiRNA-DSE* (HMMA091), *pDsU6b-BbsI-BbsI-ChiRNA DSE* (HMMA092), and *pDsU6c-BbsI-BbsI-chiRNA-DSE* (HMMA093) for transient expression of gRNAs, primer pairs HM#358/159, HM#104/158, and HM#360/160 were used to amplify the promoters of *snRNA* genes *U6a*, *U6b*, and *U6c*, respectively, with PCR condition 98 °C 3 min followed by 5 cycles of 98 °C 30 s, 66 °C 40 s, and 72 °C 1 min then 30 cycles of 98 °C 30 s, 72 °C 1 min 40 s with a final elongation 72 °C for 7 min. The promoters were then cloned into HMMA034 by megaprimer PCR cloning [60] using 30 ng of plasmid HMMA034 and 200 ng of the promoter as megaprimer in a 25 μl reaction with PCR (98 °C 3 min, [98 °C 30 s, 72 °C 2 min 30 s] 30X, 72 °C 7 min) generating plasmids HMMA088, HMMA089, and HMMA090. Finally, 250 bp of the sequence downstream of the *U6c* termination sequence was PCR amplified from genomic DNA using primer pair HM#186/187 with PCR (98 °C 3 min, [98 °C 30 s, 68 °C 30 s, 72 °C 20 s] 35X with a final elongation of 7 min at 72 °C). The amplified fragment was then cloned into HMMA088, HMMA089, and HMMA090 by megaprimer cloning as described above with annealing temperature at 68 °C.

For Cas9 recombinant protein expression, the plasmid *pET-T7-3XFlag-nls-Cas9-nls-6XHisTag* (HMMA101) was generated. The sumo part of the pET-SUMO expression vector was removed using *XhoI/NdeI* and the annealed oligos HM#152/153 were cloned introducing 2X *BsaI* sites giving rise to HMMA080. The 4.3Kb *Bbs*I*/Xba*I *3XFlag-nls-Cas9-nls* fragment was excised from HMMA066 and cloned into *BsaI* linearized HMMA080 to give rise to HMMA099. Finally, annealed oligos HM#180/181 introducing a *6XHisTag* were cloned into *FseI/BasI* digested plasmid HMMA099. Plasmid HMMA066 was generated by cloning *ClaI/HpaI* fragment *3XFlag-nls-Cas9-nls* from HMMA039 into *ClaI/HpaI* cut #1215 [20] giving rise to HMMA065 followed by cloning of annealed self-complementary oligo HM#102 into the *ClaI* site of HMMA065 to introduce 2X *BbsI* restriction sites. Cas9 protein was expressed and purified according to Paix et al. [61], and frozen at − 20 °C until needed.

The plasmid *pSLaf_Dshsp70P-Cas9-SV40_af* (HMMA056) to express Cas9 transiently was generated by cloning of the 4.2Kb *ClaI/XbaI* fragment containing insect codon optimized *Cas9* CDS with N and C terminal nuclear localization signals from plasmid #46294 (Addgene) into *ClaI/XbaI* digested pCS2-Sp6-Cas9-SV40 (Addgene: #47322) replacing the mammalian codon optimized *Cas9* CDS giving rise to HMMA039. The *Ds_hsp70* promoter was PCR amplified from genomic DNA using primer pair HM#73/75 with PCR using the following condition: 98 °C 3 min [(98C°C 30 s, 66 °C 40 s, 72 °C 1 min) 5X, (98 °C 30 s, 72 °C 1 min 40 s) 35X with a final elongation step of 7 min at 72 °C. The fragment was purified and cloned into *EcoRI/ClaI* cut #1215 [20] to give rise to HMMA052. Finally, *Cas9-SV40* was excised from HMMA039 by *ClaI/HpaI* and cloned into *ClaI/HpaI* cut HMMA052 generating HMMA056.

To generate donor plasmid HMMA134, a 3.2Kb fragment containing *PUb-nls-EGFP-SV40* was excised from #1254 [20] using *SacI/AflII* and cloned into *SacI/AflII* cut *pSLaf1108af* [59] giving rise to plasmid HMMA094. *DsRed* CDS was PCR amplified from plasmid KNE007 [58] using primer pair (HM#37/167) with PCR (98 °C 3 min followed by 35 cycles of 98 °C 30 s, 72 °C 1 min and a final elongation of 7 min at 72 °C). The fragment was phosphorylated and ligated into blunted *AflII* cut HMMA095 generating HMMA096. To change the target PAM sequence in front of *EGFP* from TGG to TGA in the repair template (Fig. 1b), PCR mutagenesis using primer pair HM#221/222 was performed (98 °C 3 min followed by 30 cycles of 98 °C 30 s, 72 °C 4 min and final elongation of 7 min at 72 °C) to give rise to HMMA097, which results in changing the second amino acid of the EGFP from valine to methionine. Finally, the 247 bp *Ds_β2t* regulatory sequence spanning − 51 to + 196 was PCR amplified using primer pair HM#285/252 with PCR conditions 98 °C 3 min [(98 °C 30 s, 60 °C 30 s, 72 °C 20 s) 5X, (98 °C 30 s, 72 °C 1 min) 30X with a final elongation step of 7 min at 72 °C. The promoter was then cloned upstream of *DsRed* in HMMA097 by megaprimer PCR cloning as described previously with annealing at 61 °C.

### Guide RNAs design, cloning, and validation

Guide RNAs were identified using the online target finder tool built by Wisconsin University (http://targetfinder.flycrispr.neuro.brown.edu/). Identified potential targets were checked against *D. suzukii* database to exclude those with off-target sites. For each potential target, two oligos, a forward and reverse, were designed and the respective overhangs were added. Oligos were ordered as normal primers without phosphorylation. The two oligos for each target were annealed at a concentration of 10 μM in a total volume of 100 μl in a heat block. The gRNAs were validated using a T7EndoI assay [62, 63]. Each *gRNA* plasmid was mixed with *Cas9* plasmid HMMA056 at a concentration of 400/500 ng/μl, respectively, and injected into 50 pre-blastoderm embryos. Ten to fifteen hatching larvae were collected in 1.5 ml Eppendorf tubes and crushed by using a pipette tip against the tube wall. Two hundred microliter of squishing buffer [19] was added and mixed well. The tubes were then incubated at 55 °C for 1 h with occasional vortexing. Tubes were then centrifuged, and 5 μl of the supernatant was used as a template in 50 μl PCR reactions using primers HM#192/69. PCR products were gel purified, quantified, and 400 ng were mixed in 1X NEB 2.1 buffer in a total volume of 19 μl. DNA was denatured, rehybridized, 0.75 μl of T7 EndoI (NEB) were added, and incubated at 37 °C for 20 min. The reactions were stopped using 2 μl of 0.25 M EDTA and run in a 1.5% agarose gel. Only one guide showed obvious digest by T7 EndoI. Wild type un-injected larvae were used as control. To generate the plasmids expressing the functional guide RNA against the identified target upstream of *DsRed* (Fig. 1b), annealed oligos HM#161/162 and HM#169/162 were cloned by golden gate [64, 65] into gRNA vectors HMMA091, HMMA092, and HMMA093 to generate p*U6a_Red1chic* HMMA102, p*U6b_Red1chi* HMMA103, and p*U6c_Red1chi* HMMA104, respectively.

### In vitro transcription of the gRNA

The functional gRNA was cloned by ligation of annealed oligos HM#162/215 into *BbsI* cut plasmid HMMA035, which was then used to generate the template for in vitro transcription by PCR using primer pair HM#84/128. In vitro transcription of *gRNA* was performed using MEGAscript® (Ambion) according to the manufacturer protocol. The reaction was allowed to proceed for 2 h at 37 °C followed by DNA template removal using 1 μl DNase I for 30 min. *gRNA* was purified using RNA clean and concentrator (Zymo Research) and the concentration was determined by nano-drop (Thermo Fisher Scientific) and stored at − 80 °C.

### Germline transformation

All embryonic injections were performed using transfection grade plasmid preparations without further precipitation steps. To generate the embryonic driver line 06_F5M2 by random *piggyBac* integration, the transformation vector HMMA006 and the helper plasmid MK006 [58] were mixed at a final concentration of 400 and 200 ng/μl respectively. To validate that the transgene represents a single integration even, we performed inversePCR as described [58] using *XhoI and EcoRI* restriction enzymes. For both the 5 and 3′ junctions, we each obtained only a single fragment, whose sequences confirmed a single integration site in the second intron of a gene referred to as *Suppressor of Under Replication* (Additional File 2: *piggyBac* insertion in *D. suzukii* line 06_F5M2).

For the transgene editing experiments using CRISPR/Cas9, DNA was mixed at a concentration of 400, 150, and 350 ng/μl for *Cas9* (HMMA056), *gRNA* (HMMA102, HMMA103, or HMMA104), and donor plasmid HMMA097, respectively. Higher concentration was used at 400, 250, and 400 ng/μl, respectively. All DNA injection mixes were prepared in 1X injection buffer (5 mM KCl, 0.1 mM NaH_2_PO_4_, pH 6.8). For RNP injection, recombinant Cas9 endonuclease, gRNA, and donor plasmid HMMA097 were mixed together at a final concentration of 300 ng/μl, 150 ng/μl, and 400 ng/μl respectively, incubated at 37 °C for 10 min for the RNP-complex formation, and injected into 90 pre-blastoderm embryos.

Injection needles were prepared as previously described [58] .To inject in *D.suzukii* embryos, the eggs have to be squeezed out of the apple agar plates individually using home-made closed-tip glass pipettes. Embryos were then de-chorionated for 3 min using generic Clorox (DanKlorix, CP GABA GmbH, Hamburg, Germany) containing 2.5% sodium hypochlorite at final concentration of 1.25% sodium hypochlorite and washed in washing buffer (100 mM NaCl, 0.02% Triton X-100) followed by thorough wash with desalted water. Embryos were then aligned on apple agar blocks and transferred to double sticky tape on a coverslip and covered by Voltalef 10S oil (VWR International, Darmstadt, Germany). Injections were performed using a Femtojet (Eppendorf, Hamburg, Germany) and a manual micromanipulator. Excessive oil was drained and the injected embryos were incubated on apple agar plates at the room temperature until hatching. Larvae were manually transferred to fly food vials. Each emerging G_0_ fly was out-crossed to 3–4 wild type individuals of the opposite sex.

### Microscopy

Screening for transgenic flies and fluorescence imaging were performed using a Leica M205 FA fluorescence stereomicroscope equipped with camera Q imaging Micropublisher 5.0 RTV (Leica Mikrosysteme Vertrieb Gmb, Wetzlar, 35,578 Germany). Transgenic flies were screened using filter sets RFP (excitation: ET546/10x, emission: ET605/70 m) or GFP-LP (excitation: ET480/40, emission: ET510 LP), respectively, and imaged using cold light (Fig. 1c) or filter sets: RFP (Figs. 1d; Fig. 3 f-h), EYFP (excitation: ET500/20, emission: ET535/30) for Fig. 1e, or GFP-LP (Fig. 3c-e).

Epifluorescence microscopy was performed using a Zeiss Imager.Z2 equipped with two cameras, Axiocam 506 mono and Axiocam 305 colour (Zeiss, 73,447 Oberkochen, Germany). The testes or the spermathecae were dissected in ice-cold PBS, fixed for 10 min in 4% formaldehyde prepared in 0.1% PBS-tween 20, permeabilized for 10 min using 1% Triton X-100 in PBS, and nuclei were stained for 10 min using DAPI (4′,6-Diamidino-2-Phenylindole, Dihydrochloride) at a concentration of 1 μg/ml. Samples were mounted in 70% glycerol and the spermathecae were broken open using dissection needles. The tissues were imaged under bright field and to observe cell nuclei and expression of DsRed, images were taken with filters for DAPI (excitation: 335–383, emission: 420–470) or DsRed (excitation: 533–558, emission: 570–640), and composed in ZEN Blue (Zeiss).

## Supplementary information

**Additional file 1: Table S1.** List of primers used.

**Additional file 2: ***piggyBac* insertion in *D. suzukii* line 06_F5M2.

## Data Availability

All data generated or analysed during this study are included in this published article and its supplementary information files.

## Abbreviations

Cas9: CRISPR associated protein 9
CRISPR: Clustered regularly interspaced short palindromic repeat
crRNA: CRISPR RNA
DIG: Digoxigenin
*Ds_lig4*: *Drosophila suzukii ligase 4*
*Ds_sryα*: *Drosophila suzukii serendipity alpha*
*Ds_tra2*: *Drosophila suzukii transformer 2*
DSB: Double strand break
DsRed: Discosoma Red
dsRNA: Double strand RNA
gRNA: Guide RNA
HDR: Homology directed repair
HEG: Homing endonuclease gene
Hsp70: Heat shock protein 70
mRNA: Messenger RNA
NHEJ: Non-homologous end joining
Orco: Odorant receptor co-receptor
PAM: Protospacer Adjacent Motif
PUb: Polyubiquitin gene
rDNA: Ribosomal deoxyribonucleic acid
RNApolIII: RNA polymerase III
RNP: Ribonucleoprotein
SIT: Sterile insect technique
snRNA: Small nuclear RNA gene
SWD: Spotted Wing *Drosophila*
*Sxl*: *Sex lethal*
TALENs: Transcription activator like endonucleases
Tc_hsp68: *Tribolium castaneum* heat shock protein 68 gene
TracrRNA: Transactivator RNA
TRE: tTA responsive element
tTA: Tetracycline controlled transactivator
ZFNs: Zinc finger nucleases
Β2tUE1: Beta-2-tubulin Upstream Element 1

## References

[1] Hauser M (2011). A historic account of the invasion of Drosophila suzukii (Matsumura) (Diptera: Drosophilidae) in the continental United States, with remarks on their identification. Pest Manag Sci.

[2] Cini A, Ioriatti C, Anfora G (2012). A review of the invasion of Drosophila suzukii in Europe and a draft research agenda for integrated pest management. Bull Insectol.

[3] Walsh DB, Bolda MP, Goodhue RE, Dreves AJ, Lee J, Bruck DJ (2011). Drosophila suzukii (Diptera: Drosophilidae): invasive pest of ripening soft fruit expanding its geographic range and damage potential. J Integr Pest Manag.

[4] Asplen MK, Anfora G, Biondi A, Choi D-S, Chu D, Daane KM (2015). Invasion biology of spotted wing Drosophila (Drosophila suzukii): a global perspective and future priorities. J Pestic Sci.

[5] Deprá M, Poppe JL, Schmitz HJ, De Toni DC, Valente VLS (2014). The first records of the invasive pest Drosophila suzukii in the south American continent. J Pestic Sci.

[6] Lavagnino NJ, Díaz BM, Cichón LI, De la Vega GJ, Garrido SA, Lago JD (2018). New records of the invasive pest Drosophila suzukii (Matsumura) (Diptera: Drosophilidae) in the south American continent. Rev Soc Entomológica Argent.

[7] Kenis M, Tonina L, Eschen R, van der Sluis B, Sancassani M, Mori N (2016). Non-crop plants used as hosts by Drosophila suzukii in Europe. J Pestic Sci.

[8] Lee JC, Bruck DJ, Curry H, Edwards D, Haviland DR, Van Steenwyk RA (2011). The susceptibility of small fruits and cherries to the spotted-wing drosophila, Drosophila suzukii. Pest Manag Sci.

[9] Lee JC, Bruck DJ, Dreves AJ, Ioriatti C, Vogt H, Baufeld P (2011). In focus: spotted wing drosophila, Drosophila suzukii, across perspectives. Pest Manag Sci.

[10] Mazzi D, Bravin E, Meraner M, Finger R, Kuske S (2017). Economic impact of the introduction and establishment of Drosophila suzukii on sweet cherry production in Switzerland. Insects..

[11] Haviland DR, Beers EH (2012). Chemical control programs for <I>Drosophila suzukii</I> that comply with international limitations on pesticide residues for exported sweet cherries. J Integr Pest Manag.

[12] Diepenbrock LM, Rosensteel DO, Hardin JA, Sial AA, Burrack HJ (2016). Season-long programs for control of Drosophila suzukii in southeastern U.S. blueberries. Crop Prot.

[13] Lee JC, Wang X, Daane KM, Hoelmer KA, Isaacs R, Sial AA (2019). Biological control of spotted-wing Drosophila (Diptera: Drosophilidae)—current and pending tactics. J Integr Pest Manag.

[14] Leach H, Van Timmeren S, Isaacs R (2016). Exclusion netting delays and reduces *Drosophila suzukii* (Diptera: Drosophilidae) infestation in raspberries. J Econ Entomol.

[15] Rendon D, Hamby KA, Arsenault-Benoit AL, Taylor CM, Evans RK, Roubos CR, et al. Mulching as a cultural control strategy for Drosophila suzukii in blueberry. Pest Manag Sci. 2019;0(ja) [cited 2019 Jun 30]. Available from: https://onlinelibrary.wiley.com/doi/abs/10.1002/ps.5512. 10.1002/ps.5512 31207075

[16] Knipling EF (1955). Possibilities of insect control or eradication through the use of sexually sterile Males1. J Econ Entomol.

[17] Krafsur ES (1998). Sterile insect technique for suppressing and eradicating insect population: 55 years and counting. J Agric Entomol.

[18] Krafsur ES, Lindquist DA (1996). Did the sterile insect technique or weather eradicate screwworms (Diptera: Calliphoridae) from Libya?. J Med Entomol.

[19] Horn C, Wimmer EA (2003). A transgene-based, embryo-specific lethality system for insect pest management. Nat Biotechnol.

[20] Schetelig MF, Caceres C, Zacharopoulou A, Franz G, Wimmer EA (2009). Conditional embryonic lethality to improve the sterile insect technique in Ceratitis capitata(Diptera: Tephritidae). BMC Biol.

[21] Ogaugwu CE, Schetelig MF, Wimmer EA (2013). Transgenic sexing system for Ceratitis capitata (Diptera: Tephritidae) based on female-specific embryonic lethality. Insect Biochem Mol Biol.

[22] Yan Y, Scott MJ (2015). A transgenic embryonic sexing system for the Australian sheep blow fly Lucilia cuprina. Sci Rep.

[23] Scolari F, Schetelig MF, Bertin S, Malacrida AR, Gasperi G, Wimmer EA (2008). Fluorescent sperm marking to improve the fight against the pest insect Ceratitis capitata (Wiedemann; Diptera: Tephritidae). N Biotechnol.

[24] Smith RC, Walter MF, Hice RH, O’Brochta DA, Atkinson PW (2007). Testis-specific expression of the ?2 tubulin promoter of Aedes aegypti and its application as a genetic sex-separation marker. Insect Mol Biol.

[25] Zimowska GJ, Nirmala X, Handler AM (2009). The β2-tubulin gene from three tephritid fruit fly species and use of its promoter for sperm marking. Insect Biochem Mol Biol.

[26] Catteruccia F, Benton JP, Crisanti A (2005). An Anopheles transgenic sexing strain for vector control. Nat Biotechnol.

[27] Ishino Y, Shinagawa H, Makino K, Amemura M, Nakata A (1987). Nucleotide sequence of the iap gene, responsible for alkaline phosphatase isozyme conversion in Escherichia coli, and identification of the gene product. J Bacteriol.

[28] Makarova KS, Grishin NV, Shabalina SA, Wolf YI, Koonin EV (2006). No title found. Biol Direct.

[29] Barrangou R, Fremaux C, Deveau H, Richards M, Boyaval P, Moineau S (2007). CRISPR provides acquired resistance against viruses in prokaryotes. Science..

[30] Garneau JE, Dupuis M-È, Villion M, Romero DA, Barrangou R, Boyaval P (2010). The CRISPR/Cas bacterial immune system cleaves bacteriophage and plasmid DNA. Nature..

[31] Jinek M, Chylinski K, Fonfara I, Hauer M, Doudna JA, Charpentier E (2012). A programmable dual-RNA-guided DNA endonuclease in adaptive bacterial immunity. Science..

[32] Bassett AR, Tibbit C, Ponting CP, Liu J-L (2013). Highly efficient targeted mutagenesis of Drosophila with the CRISPR/Cas9 system. Cell Rep.

[33] Hwang WY, Fu Y, Reyon D, Maeder ML, Tsai SQ, Sander JD (2013). Efficient genome editing in zebrafish using a CRISPR-Cas system. Nat Biotechnol.

[34] Platt RJ, Chen S, Zhou Y, Yim MJ, Swiech L, Kempton HR (2014). CRISPR-Cas9 Knockin mice for genome editing and Cancer modeling. Cell..

[35] Hall B, Cho A, Limaye A, Cho K, Khillan J, Kulkarni AB (2018). Genome editing in mice using CRISPR/Cas9 technology. Curr Protoc Cell Biol.

[36] Gratz SJ, Cummings AM, Nguyen JN, Hamm DC, Donohue LK, Harrison MM (2013). Genome engineering of *Drosophila* with the CRISPR RNA-guided Cas9 nuclease. Genetics..

[37] Li M, Bui M, Yang T, Bowman CS, White BJ, Akbari OS (2017). Germline Cas9 expression yields highly efficient genome engineering in a major worldwide disease vector, *Aedes aegypti*. Proc Natl Acad Sci.

[38] Li M, Akbari OS, White BJ (2018). Highly Efficient Site-Specific Mutagenesis in Malaria Mosquitoes Using CRISPR. G3.

[39] Jiang J, Zhang L, Zhou X, Chen X, Huang G, Li F (2016). Induction of site-specific chromosomal translocations in embryonic stem cells by CRISPR/Cas9. Sci Rep.

[40] Iwata S, Yoshina S, Suehiro Y, Hori S, Mitani S (2016). Engineering new balancer chromosomes in *C. elegans* via CRISPR/Cas9. Sci Rep.

[41] Port F, Bullock SL (2016). Augmenting CRISPR applications in Drosophila with tRNA-flanked sgRNAs. Nat Methods.

[42] Port F, Chen H-M, Lee T, Bullock SL (2014). Optimized CRISPR/Cas tools for efficient germline and somatic genome engineering in Drosophila. Proc Natl Acad Sci.

[43] Li F, Scott MJ (2016). CRISPR/Cas9-mediated mutagenesis of the white and sex lethal loci in the invasive pest, Drosophila suzukii. Biochem Biophys Res Commun.

[44] Kalajdzic P, Schetelig MF (2017). CRISPR/Cas-mediated gene editing using purified protein in *Drosophila suzukii*. Entomol Exp Appl.

[45] Li J, Handler AM (2017). Temperature-dependent sex-reversal by a transformer-2 gene-edited mutation in the spotted wing drosophila, Drosophila suzukii. Sci Rep.

[46] Karageorgi M, Bräcker LB, Lebreton S, Minervino C, Cavey M, Siju KP (2017). Evolution of multiple sensory systems drives novel egg-laying behavior in the fruit Pest Drosophila suzukii. Curr Biol.

[47] Eckermann KN, Dippel S, KaramiNejadRanjbar M, Ahmed HM, Curril IM, Wimmer EA (2014). Perspective on the combined use of an independent transgenic sexing and a multifactorial reproductive sterility system to avoid resistance development against transgenic sterile insect technique approaches. BMC Genet.

[48] Kemphues J, Kaufman C, Raff A, Raff C (1982). The testis-specific P-tubulin subunit in Drosophila melanogaster has multiple functions in spermatogenesis. Cell..

[49] Michiels F, Gasch A, Kaltschmidt B, Renkawitz-Pohl R (1989). A 14 bp promoter element directs the testis specificity of the Drosophila 32 tubulin gene. EMBO J.

[50] Michiels F, Wolk A, Renkawitz-Pohl R (1991). Further sequence requirements for male germ cell-specific expression under the control of the 14 bp promoter element (B2UE1) of the Drosophila B2 tubulin gene. Nucleic Acids Res.

[51] Galizi R, Doyle LA, Menichelli M, Bernardini F, Deredec A, Burt A (2014). A synthetic sex ratio distortion system for the control of the human malaria mosquito. Nat Commun.

[52] Schetelig MF, Handler AM (2012). A transgenic embryonic sexing system for Anastrepha suspensa (Diptera: Tephritidae). Insect Biochem Mol Biol.

[53] Kondo S, Ueda R (2013). Highly improved gene targeting by Germline-specific Cas9 expression in Drosophila. Genetics..

[54] Shoji W, Sato-Maeda M (2008). Application of heat shock promoter in transgenic zebrafish. Dev Growth Differ.

[55] Schulte C, Leboulle G, Otte M, Grünewald B, Gehne N, Beye M (2013). Honey bee promoter sequences for targeted gene expression: honey bee promoter analysis. Insect Mol Biol.

[56] Lécuyer E, Gerst JE (2011). High Resolution Fluorescent In Situ Hybridization in Drosophila. RNA Detection and Visualization.

[57] Zimmerman SG, Peters NC, Altaras AE, Berg CA (2013). Optimized RNA ISH, RNA FISH and protein-RNA double labeling (IF/FISH) in Drosophila ovaries. Nat Protoc.

[58] Eckermann KN, Ahmed HMM, KaramiNejadRanjbar M, Dippel S, Ogaugwu CE, Kitzmann P (2018). Hyperactive piggyBac transposase improves transformation efficiency in diverse insect species. Insect Biochem Mol Biol.

[59] Horn C, Wimmer EA (2000). A versatile vector set for animal transgenesis. Dev Genes Evol.

[60] Ulrich A, Andersen KR, Schwartz TU (2012). Exponential Megapriming PCR (EMP) Cloning—Seamless DNA Insertion into Any Target Plasmid without Sequence Constraints. Isalan M, editor. PLoS One.

[61] Paix A, Folkmann A, Rasoloson D, Seydoux G (2015). High efficiency, homology-directed genome editing in *Caenorhabditis elegans* using CRISPR-Cas9 Ribonucleoprotein complexes. Genetics..

[62] Vouillot L, Thélie A, Pollet N (2015). Comparison of T7E1 and Surveyor Mismatch Cleavage Assays to Detect Mutations Triggered by Engineered Nucleases. G3amp58 Gene Genom Genet.

[63] Huang MC, Cheong WC, Lim LS, Li M-H (2012). A simple, high sensitivity mutation screening using Ampligase mediated T7 endonuclease I and surveyor nuclease with microfluidic capillary electrophoresis. Electrophoresis..

[64] Engler C, Kandzia R, Marillonnet S (2008). A one pot, one step, precision cloning method with high throughput capability. El-Shemy HA, editor. PLoS One.

[65] Engler C, Gruetzner R, Kandzia R, Marillonnet S (2009). Golden gate shuffling: A one-pot dna shuffling method based on type iis restriction enzymes. Peccoud J, editor. PLoS One.

