# Supplementary material for: Improvement and use of CRISPR/Cas9 to engineer a sperm-marking strain for the invasive fruit pest Drosophila suzukii
Source: BMC Biotechnol. 2019 Dec 5;19:85. doi: 10.1186/s12896-019-0588-5 (PMC6896403; doi:10.1186/s12896-019-0588-5)
Supplement: Supplementary file 2 — Additional file 2: piggyBac insertion in D. suzukii line 06_F5M2. [file 12896_2019_588_MOESM2_ESM.docx]

**Additional file 2: *piggyBac* insertion in *D. suzukii* line 06_F5M2**

*piggyBac* insertion in the second intron of a gene referred to as *Suppressor of Under-Replication* (*SuUR*). Underlined are the restriction sites for *EcoR*I and *Xho*I, respectively. The bold ***TTAA*** sequence represents the site of *piggyBac* integration, which was duplicated.

TTCGCGAATTCTGCATCCTGAACGATGAGAGTGGCCTGGGTAAACTGGCCACGGTGGCGGCACTTCTCAGTGCCCTAGATCCCGCCAAGAAAACTCTCATTGTGCTGCAGAACGACGAGCAACTGCTCGCTGGTTGGCGGTTCCATCTGGACACACTCACGAACCTGCAGGTGTACACCATTCAAGGAGTCCAAGGTAATGCTCTGCGTTTAATGATATTCTTGGATTTCTATGTGGATTAGAATTTGGAAAACACCATGTTCTGATATTTTTATACTTCTCCTAAATCAACATTCCTTGATAGTTAGTTTCGAAGAATGAATCTCWCTGCAGCGTAAAATGMYTATATTTTGAAAACCACAAAATTGATCAGTTTTTTATTTTGTGTGTACAATTTCGCAATTAAACGAAAGTAGTGGGTTCCATTTAAGGGATCTGTTAAAATTTGATTTTACCAACTTTATTTTGCTATACGGTCTGAACCATTTTAAATAATTTTTTATTATAGTTTAGTTTATAGTTTTATTTACAAAATAAATAATGAATATTCTTTAAACTGCTAAAACAATTTGAAAAATTAACATTATTTCCATATAATCAATTTTTAAAACGAAAACTTGT***TTAA_5’piggyBac3’_TTAA***TTAAGTCTTGTATAAACCTTATCAAAGAACTATATATTTTTATTCGGAAACTAACAGTTTGTTTTACCTCCTTTACAGACACCACAGACTCCCCGCACAACGTTTACCTGGCAAAGTGGAGCCAGTTACGCAGCATTGGAGATCTCAGTCGCCTCAAGTTCGACTACGTTCTGGTAGACAATCGGGGCCACACGCTGAACAACAGCTTCTGCACATCAATGCTTCTCAAGCATTTTGAGGGAAGGGTAAACATTCTTATCTCCAGTGTTGACATTACGGTGAGGCAATGCACCCATAAGTGGTTCACATGGAGCTTTATTTAGTTTGTTTCCCTATCTGCAGTCAGACGTAAGGTTGCTGTTCATGTTTTGCGGTTGGGCGGGCGCCTGGAGCATCAGTATCGGAGCTTCCAGAGCTTCGACCGCAAGTTCCATTTGCCAGATCCAAAGGAGGTCTTTAGCAAGCGTATAGATCTCGAGAT
